# Computational and theoretical insights into the homeostatic response to the decreased cell size of midbrain dopamine neurons

**DOI:** 10.14814/phy2.14709

**Published:** 2021-01-23

**Authors:** Francisco Arencibia‐Albite, Carlos A. Jiménez‐Rivera

**Affiliations:** ^1^ Department of Physiology University of Puerto Rico San Juan Puerto Rico; ^2^ Department of Natural Sciences University of Sacred Heart San Juan Puerto Rico

**Keywords:** capacitance, cell size, computational modeling, dopamine neurons

## Abstract

Midbrain dopamine neurons communicate signals of reward anticipation and attribution of salience. This capacity is distorted in heroin or cocaine abuse or in conditions such as human mania. A shared characteristic among rodent models of these behavioral disorders is that dopamine neurons in these animals acquired a small size and manifest an augmented spontaneous and burst activity. The biophysical mechanism underlying this increased excitation is currently unknown, but is believed to primarily follow from a substantial drop in K^+^ conductance secondary to morphology reduction. This work uses a dopamine neuron mathematical model to show, surprisingly, that under size diminution a reduction in K^+^ conductance is an adaptation that attempts to decrease cell excitability. The homeostatic response that preserves the intrinsic activity is the conservation of the ion channel density for each conductance; a result that is analytically demonstrated and challenges the experimentalist tendency to reduce intrinsic excitation to K^+^ conductance expression level. Another unexpected mechanism that buffers the raise in intrinsic activity is the presence of the ether‐a‐go‐go‐related gen K^+^ channel since its activation is illustrated to increase with size reduction. Computational experiments finally demonstrate that size attenuation results in the paradoxical enhancement of afferent‐driven bursting as a reduced temporal summation indexed correlates with improved depolarization. This work illustrates, on the whole, that experimentation in the absence of mathematical models may lead to the erroneous interpretation of the counterintuitive aspects of empirical data.

## Graphical abstract

This article uses computational and analytical methods to illustrate that a reduction in potassium conductance, under cell size diminution, dampens the expected rise in intrinsic cell spiking and not the opposite. We demonstrate that the neuronal firing rate and spike waveform are, in effect, insensitive to changes in cell size as long as the number of channels per unit of membrane area is preserved. This work illustrates, on the whole, that experimentation in the absence of sound mathematical models may lead to an erroneous interpretation of reality.

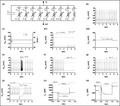

## 1 INTRODUCTION

Midbrain dopamine (DA) neurotransmission is essential for the control of voluntary movement (Sharples et al., 2014), motivation (Bromberg‐Martin et al., 2010) and is hypothesized to be a key mediator of addictive behaviors (Kalivas & Volkow, 2007; Wise, 2004). DA releasing neurons involved in motor function are located in the substantia nigra pars compacta and their axons mainly innervate the dorsal striatum (Smith & Kieval, 2000), whereas those associated with limbic and cognitive functions are found in the ventral tegmental area (VTA), and primarily project to the nucleus accumbens in the ventral striatum and the prefrontal cortex (Neuhoff et al., 2002). In the brain slice preparation, dopaminergic neurons express regular or irregular single‐spike spontaneous activity in the range 1–7 Hz (Grace & Onn, 1989; Ping & Shepard, 1996). In freely moving (Hyland et al., 2002) and anesthetized (Lee et al., 2004) rats, however, afferent‐driven bursts of action potentials are superimposed onto this autonomous background. Such a burst signal produces a transient increase in DA concentration greater than that of intrinsically evoked single‐spike activity (Chergui et al., 1996; Wightman & Heien, 2006). In animal behavior studies the burst discharge is observed at the moment the animal receives an unpredicted reward or is unexpectedly presented with the opportunity to initiate an action that ends in reward (Schultz, 1998, 2002). DA neurons are, therefore, thought to convey signals of reward anticipation and attribution of salience (Schultz, 2006).

The capacity of DA neurons to encode and predict reward may be substantially transformed during chronic exposure to drugs of abuse or in conditions such as human mania. In mice and rats repeated morphine, heroin or cocaine administration decreases the size of VTA DA neurons (Arencibia‐Albite et al., 2012, 2017; Mazei‐Robison et al., 2011; Russo et al., 2006). In the case of morphine and heroin, the emergence of a diminished morphology correlates with the development of reward tolerance, which may help to explain the escalation in drug use (Russo et al., 2006). Moreover, mice with a mutation in the Clock gene have smaller VTA DA neurons and manifest behavioral measures of mood, anxiety, activity, and reward that are remarkably similar to bipolar patients in the manic state (Coque et al., 2011; Mcclung et al., 2005; Roybal et al., 2007). These genetically altered mice also show hyperactive and hyperhedonic traits that are abolished as normal neuronal dimensions are rescued with lithium treatment (Coque et al., 2011). A unifying attribute among drug‐treated mice and Clock mutants is that their smaller VTA DA neurons have an increased autonomous firing and bursting, in contrast, to similar neurons from wild‐type littermates (Coque et al., 2011; Mazei‐Robison et al., 2011; Mcclung et al., 2005).

The biophysical mechanisms underlying the increased excitation that appears in a DA cell with an undersized structure is currently poorly understood. In order to explain the latter, most researchers will make use of the well‐established experimental rule that the augmentation of the membrane conductance to K^+^ inhibits cell activity (Arencibia‐Albite et al., 2007), while its reduction enhances excitability (Hopf et al., 2007; Ji et al., 2012). In view of this empirical principle, Mazei‐Robison et al. (Mazei‐Robison et al., 2011) showed that K^+^ channel expression is down‐regulated in morphine‐exposed mice and concluded that such an event is at least one of the main culprits for the augmented cell firing. As a consequence, if this reasoning is correct then the appropriate homeostatic response that recovers normal intrinsic excitability is the return of each altered conductance to its natural pretreatment state, that is, the size of each distinct ion channel population in the neuronal membrane should be equal to that of the untreated condition. The present report uses numerical simulations to show that, surprisingly, this is not the case. In striking contrast to experimental reasoning, the computational modeling illustrates that the reduction in K^+^ conductance, in the presence of diminished cell size, is a compensatory mechanism that dampens the augmented firing and not an event that drives further excitation as qualitative inspection of experimental data may suggest. Simulations also illustrate that a smaller DA neuron will express a reduced synaptic summation index; yet, it will burst at a much faster rate and with a higher spike count per burst than a normal size cell when exposed to the same train of excitatory post‐synaptic currents.

## 2 MATERIALS AND METHODS

The model used in this study is adapted from existing models of DA neurons (Komendantov et al., 2004; Kuznetsova et al., 2010; Yu & Canavier, 2015; Yu et al., 2014). Komendantov et al. (Komendantov et al., 2004) represent the typical DA neuron with thirteen compartments, including one soma, four proximal dendrites, and eight distal dendrites. Symmetry considerations allow the thirteen‐compartment model to be equivalent to a three‐compartment model: one soma, one proximal dendrite, and one distal dendrite (Kuznetsova et al., 2010; Yu et al., 2014). This not only simplifies the model's computational implementation but also captures the fundamental electrophysiological properties of multi‐compartmental models that expressed a realistic DA cell morphology (Kuznetsova et al., 2010). The DA neuron model used in this study consists, therefore, of these three compartments.

**FIGURE 1:**
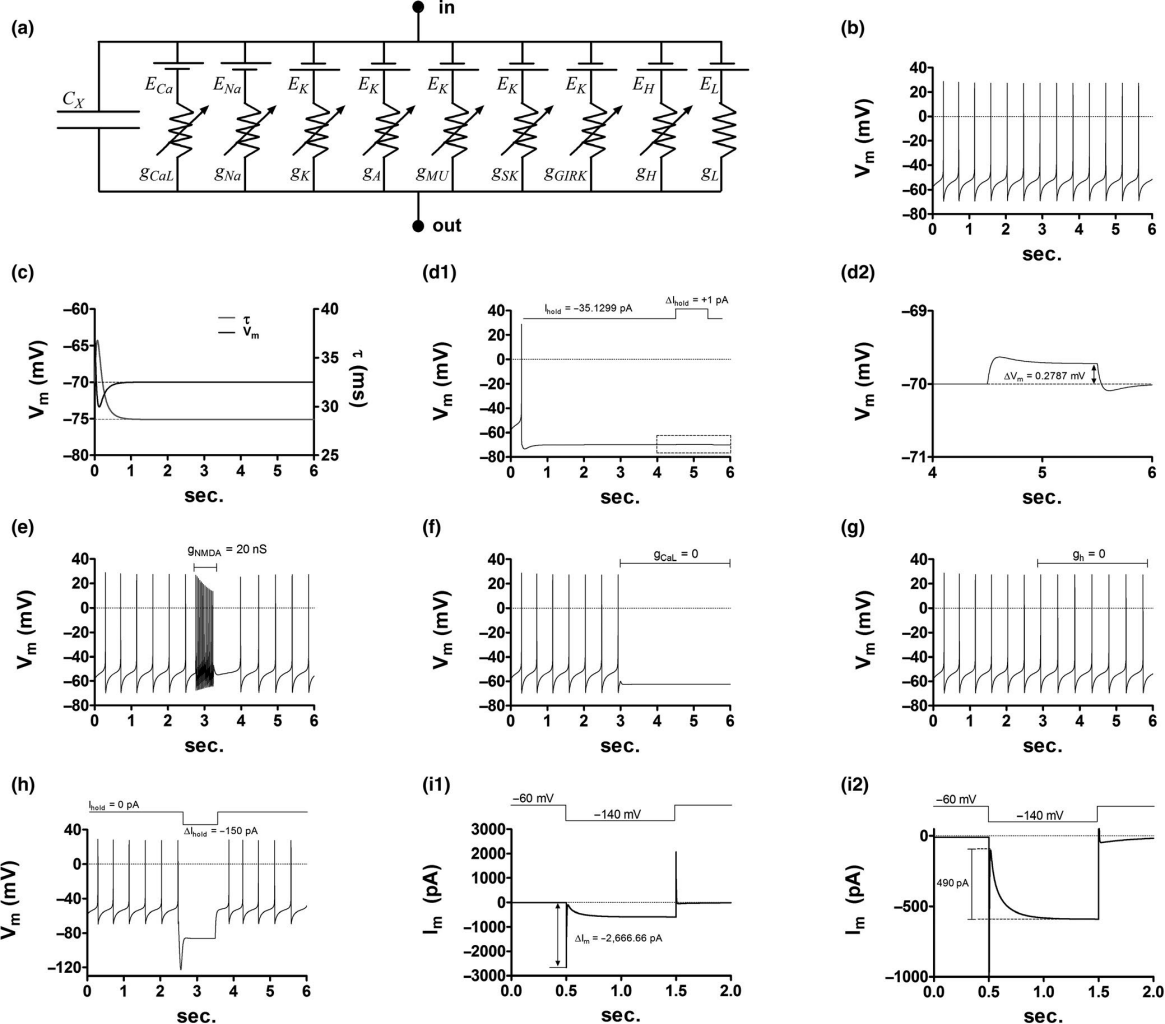
DA neuron model electrophysiology. (a) All compartments in the model are equivalent to the depicted circuit. Na^+^ and Ca^++^ currents are inward whereas K^+^ currents are outward. The h and leak currents reverse direction during the action potential time course. For further details see Materials and Methods. (b) The model's soma compartment exhibits a slow pacemaker firing at 2.16 Hz under control conditions. (c) Measurement of the model's membrane time constants when the soma compartment is held at −70 mV. (d1) Input resistance test performed at the soma compartment. (d2) The graph expands the content of the dashed box in c1. (e) The model's burst response as elicited by NMDA channels placed at the soma compartment. (f) The soma spontaneous activity in the model is driven by the L‐type Ca^++^ conductance (*g*_CaL_); *g*_CaL_ was set to zero in all compartments. (g) The inhibition of the h‐conductance (*g*_H_) has no impact on the model's pacemaker activity; *g*_H_ was set to zero in all compartments. (h) Depolarization sag response measured at the soma compartment. (i1) Simulated voltage‐clamp whole‐cell recording. The pipette was placed at the soma compartment. The access resistance is 30 MΩ. (i2) Zoom in on the recording in i1.

The model compartments are treated as cylinders with defined length (L) and diameter (d) whose equivalent circuits are as shown in Figure 1a. The current balance equations for compartments are (S = soma, P = proximal dendrite, D = distal dendrite):

$$\begin{array}{cc} C_{S}\frac{dV_{S}}{dt}= & ‐I_{\text{Na,S}}‐I_{K,S}‐I_{\text{SK},S}‐I_{A,S}‐I_{\text{MU},S}‐I_{\text{GIRK},S}\\ & ‐I_{\text{CaL},S}‐I_{h,S}‐I_{L,S}+g_{\text{SP}}\left(V_{P}‐V_{S}\right) \end{array}$$

$$\begin{array}{cc} C_{P}\frac{dV_{P}}{dt}= & ‐I_{\text{Na},P}‐I_{K,P}‐I_{\text{SK},P}‐I_{A,P}\\ & ‐I_{\text{MU},P}‐I_{\text{GIRK},P}‐I_{\text{CaL},P}‐I_{h,P}‐I_{L,P}\\ & +g_{\text{SP}}\left(V_{S}‐V_{P}\right)+g_{\text{PD}}\left(V_{D}‐V_{P}\right) \end{array}$$

$$\begin{array}{cc} C_{D}\frac{dV_{D}}{dt}= & ‐I_{\text{Na},D}‐I_{K,D}‐I_{\text{SK},D}‐I_{A,D}‐I_{\text{MU},D}‐I_{\text{GIRK},D}\\ & ‐I_{\text{CaL},D}‐I_{h,D}‐I_{L,D}+g_{\text{PD}}\left(V_{P}‐V_{D}\right) \end{array}$$

where *I*_Na,X_ is the fast Na^+^ current, *I*_K,X_ is the delayed rectifier K^+^ current, *I*_SK,X_ is the small K^+^ conductance current, *I*_A,X_ is the transient outward K^+^ current, *I*_MU,X_ the muscarinic K^+^ current, *I*_GIRK,X_ is the G‐protein‐gated inward rectifying K^+^ current, *I*_Cal,X_ is the non‐inactivating L‐type Ca^++^ current, *I*_h,X_ is the hyperpolarization‐activated cation current, *I*_L,X_ is the leak current, *g*_SP_(*V*_P_ − *V*_S_) and *g*_SP_(*V*_S_ − *V*_P_) are the coupling currents between the soma and the proximal dendrite, while *g*_PD_(*V*_D_ − *V*_P_) and *g*_PD_(*V*_P_ − *V*_D_) couple the proximal and distal dendrites. The length, diameter, surface area (Sa), and capacitance (*C*) for each compartment are:

$$\begin{array}{cc} & L_{S}=25\;\mu m,d_{S}=15\;\mu m,{\text{Sa}}_{S}\approx 1,178\;{\mu m}^{2},\\ & C_{S}=20\;\text{pF}\;\left(1.7\;\mu F/{\text{cm}}^{2}\right) \end{array}$$

$$\begin{array}{cc} & L_{P}=150\;\mu m,d_{P}=3\;\mu m,{\text{Sa}}_{P}\approx 1,414\;{\mu m}^{2},\\ & C_{P}=30\text{pF}\;\left(2.122\;\mu F/{\text{cm}}^{2}\right) \end{array}$$

$$\begin{array}{cc} & L_{D}=350\;\mu m,d_{D}=1.5\;\mu m,{\text{Sa}}_{D}\approx 1,649\;{\mu m}^{2},\\ & C_{P}=30\;\text{pF}\;\left(1.82\;\mu F/{\text{cm}}^{2}\right) \end{array}$$

Ionic currents in the model obey the following set of equations. Parameters values in the equations are listed as they appear in the numerical algorithm. The units in the programmed iterations were mV for membrane potential, nS for conductance, pA for current, and ms for time.

Fast Na^+^ current (*I*_Na_):

$$\begin{array}{cc} & I_{\text{Na,X}}=g_{\text{Na,X}}\;m^{3}p\;h_{\text{SS}}\left(V_{X}‐E_{\text{Na}}\right),\\ & g_{\text{Na,S}}=g_{\text{Na,P}}=g_{\text{Na,D}}=450\;\text{nS}\\ & E_{\text{Na}}=40\;\text{mV} \end{array}$$

$$\frac{dm}{dt}=\alpha_{m}\left(V_{X}\right)\left(1‐m\right)‐\beta_{m}\left(V_{X}\right)m$$

$$\frac{dp}{dt}=\alpha_{p}\left(V_{X}\right)\left(1‐p\right)‐\beta_{p}\left(V_{X}\right)p$$

$$\frac{dh_{\text{SS}}}{dt}=\frac{h_{\text{SS},\infty }\left(V_{X}\right)‐h_{\text{SS}}}{\tau_{h_{\text{SS}}}\left(V_{X}\right)}$$

$$\alpha_{p}\left(V_{X}\right)=0.07\;\exp\left[‐\left(V_{X}+40\right)/20\right]$$

$$\alpha_{m}\left(V_{X}\right)=\frac{0.1V_{X}+2.5}{1‐\exp\left[‐\left(0.1V_{X}+2.5\right)\right]}$$

$$\beta_{p}\left(V_{X}\right)=\frac{1}{1+\exp\left[‐\left(0.1V_{X}+1.4\right)\right]}$$

$$\beta_{m}\left(V_{X}\right)=4\exp\left[‐\left(V_{X}+50\right)/18\right]$$

$$h_{\text{SS},\infty }\left(V_{X}\right)=\frac{1}{1+\exp\left[V_{X}+45\right]}$$

$$\tau_{h_{\text{SS}}}\left(V_{X}\right)=20+\frac{580}{1+\exp\left[V_{X}\right]}$$

Delayed rectifier K^+^ current (*I*_K_):

$$\begin{array}{cc} & I_{\text{K,X}}=g_{\text{K,X}}\;n^{4}\left(V_{X}‐E_{K}\right),\;g_{\text{K,S}}=225\;\text{nS},\\ & g_{\text{K,P}}=g_{\text{K,D}}=175\;\text{nS}\;E_{K}=‐78\;\text{mV} \end{array}$$

$$\frac{dn}{dt}=\alpha_{n}\left(V_{X}\right)\left(1‐n\right)‐\beta_{n}\left(V_{X}\right)n$$

$$\alpha_{n}\left(V_{X}\right)=\frac{0.01\left(V_{X}+34\right)}{1‐\exp\left[‐0.2\left(V_{X}+34\right)\right]}$$

$$\beta_{n}\left(V_{X}\right)=0.125\exp\left[‐\left(V_{X}+40\right)/80\right]$$

Small K^+^ conductance current (*I*_SK_):

$$\begin{array}{cc} & I_{\text{SK,X}}=\frac{g_{\text{SK,X}}\left(V_{X}‐E_{K}\right)}{1+{\left(K_{\text{SK}}/{\left[{\text{Ca}}^{++}\right]}_{\text{in,X}}\right)}^{4}},\\ & g_{\text{SK,S}}=g_{\text{SK,P}}=0.25\;\text{nS},\;g_{\text{SK,D}}=0.3\;\text{nS}\\ & K_{\text{SK}}=0.00019\;\text{mM} \end{array}$$

$$\frac{d{\left[{\text{Ca}}^{++}\right]}_{\text{in,X}}}{dt}=‐\frac{f_{\text{Ca}}I_{\text{CaL,X}}}{2F\cdot \text{vol}}‐\beta_{\text{Ca}}\left({\left[{\text{Ca}}^{++}\right]}_{\text{in,X}}‐{\left[{\text{Ca}}^{++}\right]}_{\min}\right)$$

where *f*_Ca_ = 0.001; *F* = 96,520 C/mol; vol = 0.0117 fL; *β*_Ca_ = 0.05; [Ca^++^]min = 0.00001 mM.

Transient outward K^+^ current (*I*_A_):

$$I_{\text{A,X}}=g_{\text{A,X}}\;r\;q^{3}\left(V_{X}‐E_{K}\right),\;g_{\text{A,S}}=3\;\text{nS},\;g_{\text{A,P}}=g_{\text{A,D}}=4\;\text{nS}$$

$$\frac{dr}{dt}=\frac{r_{\infty }\left(V_{X}\right)‐r}{20}$$

$$\frac{dq}{dt}=\frac{q_{\infty }\left(V_{X}\right)‐q}{15}$$

$$r_{\infty }\left(V_{X}\right)=\frac{1}{1+\exp\left[\left(V_{X}+63/4\right)\right]}$$

$$q_{\infty }\left(V_{X}\right)=\frac{1}{1+\exp\left[‐\left(V_{S}+43\right)/24\right]}$$

Muscarinic K^+^ current (*I*_MU_):

$$\begin{array}{cc} & I_{\text{MU,X}}=g_{\text{MU,X}}\;\mu \left(V_{X}‐E_{K}\right),\\ & g_{\text{MU,S}}=1.5\;\text{nS},\;g_{\text{MU,P}}=1.8\;\text{nS},\;g_{\text{MU,D}}=2.1\;\text{nS} \end{array}$$

$$\frac{d\mu }{dt}=\alpha_{\mu }\left(V_{X}\right)\left(1‐\mu \right)‐\beta_{\mu }\left(V_{X}\right)\mu$$

$$\alpha_{\mu }\left(V_{X}\right)=\frac{0.02}{1+\exp\left[‐\left(V_{X}+20\right)/5\right]}$$

$$\beta_{\mu }\left(V_{X}\right)=0.01\exp\left[‐\left(V_{X}+43\right)/18\right]$$

G‐protein gated inward rectifying K^+^ current (*I*_GIRK_):

$$\begin{array}{cc} & I_{\text{GIRK,X}}=\frac{g_{\text{GIRK,X}}\left(V_{X}‐E_{K}\right)}{1+\exp\left[\left(V_{X}+45\right)/20\right]},\\ & g_{\text{GIRK,S}}=0.012\;\text{nS},\;g_{\text{GIRK,P}}=0.0144\;\text{nS},\;g_{\text{GIRK,D}}=0.0168\;\text{nS} \end{array}$$

L‐type Ca^++^ current (*I*_CaL_):

$$\begin{array}{cc} & I_{\text{CaL,X}}=g_{\text{CaL,X}}l\left(V_{X}‐E_{\text{Ca}}\right),\\ & g_{\text{CaL,S}}=0.14875\;\text{nS},\;g_{\text{CaL,P}}=0.2125\;\text{nS},\;g_{\text{CaL,D}}=0.2975\;\text{nS},\\ & E_{\text{Ca}}=70\;\text{mV} \end{array}$$

$$\frac{dl}{dt}=\frac{l_{\infty }\left(V_{X}\right)‐l}{\tau_{l}\left(V_{X}\right)}$$

$$l_{\infty }\left(V_{X}\right)=\frac{1}{1+\exp\left[‐\left(V_{X}+42\right)/12\right]}$$

$$\tau_{l}\left(V_{X}\right)=5\exp\left[‐{\left(V_{X}+70\right)}^{2}/625\right]+0.25$$

Hyperpolarization‐activated cation current (*I*_h_):

$$\begin{array}{cc} & I_{\text{h,X}}=g_{\text{h,X}}\;h_{X}\left(V_{X}‐E_{h}\right);\\ & g_{\text{h,S}}=2.5\;\text{nS},\;g_{\text{h,P}}=3\;\text{nS}\;g_{\text{h,D}}=3.5\;\text{nS},\\ & E_{h}=‐53\;\text{mV} \end{array}$$

$$\frac{dh_{X}}{dt}=\frac{h_{\infty ,X}\left(V_{X}\right)‐h_{X}}{\tau_{\text{h,X}}\left(V_{X}\right)}$$

$$h_{\infty ,X}\left(V_{X}\right)=\frac{1}{1+\exp\left[\left(V_{X}+90\right)/8\right]}$$

$$\tau_{\text{h,X}}\left(V_{X}\right)=\frac{425\exp\left(0.075\left(V_{X}+112\right)\right)}{1+\exp\left(0.083\left(V_{X}+112\right)\right)}$$

Ether‐a‐go‐go related gen K^+^ (ERG) current (*I*_ERG_):

$$\begin{array}{cc} & I_{\text{ERG,X}}=g_{\text{ERG,X}}o\left(V_{X}‐E_{K}\right);\\ & g_{\text{ERG,S}}=0.3\;\text{nS},\;g_{\text{ERG,P}}=0.36\;\text{nS}\;g_{\text{ERG,D}}=0.42\;\text{nS} \end{array}$$

$$\frac{do}{dt}=\alpha_{o}\left(1‐o‐i\right)+\beta_{i}i‐o\left(\alpha_{i}+\beta_{o}\right)$$

$$\frac{di}{dt}=\alpha_{i}o‐\beta_{i}i$$

$$\alpha_{o}\left(V_{X}\right)=0.0036\exp\left(0.0759V_{X}\right)$$

$$\beta_{o}\left(V_{X}\right)=1.2523\times 10^{‐5}\exp\left(‐0.0671V_{X}\right)$$

$$\alpha_{i}\left(V_{X}\right)=92.11\exp\left(0.1189V_{X}\right)$$

$$\beta_{i}\left(V_{X}\right)=12.6\exp\left(0.0733V_{X}\right)$$

Leak currents (*I*_L_):

$$\begin{array}{cc} & I_{\text{L,X}}=g_{\text{L,X}}\left(V_{x}‐E_{X}\right),\\ & g_{\text{L,S}}=0.35\;\text{nS},\;g_{\text{L,P}}=g_{\text{L,D}}=0.65\;\text{nS},\\ & E_{S}=E_{P}=E_{D}=‐58\;\text{mV} \end{array}$$

The coupling conductances between compartments are:

$$\begin{array}{cc} & g_{\text{SP}}=\frac{\pi \times 10^{‐4}}{2R_{i}\left[\frac{L_{S}}{d_{S}^{2}}+\frac{L_{P}}{d_{P}^{2}}\right]}=234\;\text{nS},\\ & g_{\text{PD}}=\frac{\pi \times 10^{‐4}}{2R_{i}\left[\frac{L_{P}}{d_{P}^{2}}+\frac{L_{D}}{d_{D}^{2}}\right]}=22.8\;\text{nS},\\ & R_{i}=40\Omega ‐\text{cm} \end{array}$$

### 2.1 Simulations in cell size alteration

In order to simulate reductions in cell size we assumed, as a first approximation, that a neuron with a decreased size preserves its relative dimensions. Thus, as cell capacitance decreases the ratio of length to diameter in each compartment is kept constant. This signifies, as an example, that if cell capacitance is decreased by 36% the coupling conductances between compartments are adjusted using the following new diameter and length values

$$L_{\text{new}}=\sqrt{1‐\frac{36}{100}}L_{\text{old}}=\sqrt{0.64}L_{\text{old}}=0.8L_{\text{old}}$$

$$d_{\text{new}}=\sqrt{1‐\frac{36}{100}}d_{\text{old}}=\sqrt{0.64}d_{\text{old}}=0.8d_{\text{old}}$$

Since

$$\begin{array}{cc} C_{\text{compartment},\;\;\text{new}} & =\left(1‐\frac{36}{100}\right){\hat{C}}_{m}\cdot {\text{Sa}}_{\text{compartment},\;\;\text{old}}\\ & \;=\left(1‐\frac{36}{100}\right){\hat{C}}_{m}\pi d_{\text{old}}L_{\text{old}}={\hat{C}}_{m}\;\pi \left\{\sqrt{1‐\frac{36}{100}}d_{\text{old}}\right\}\left\{\sqrt{1‐\frac{36}{100}}L_{\text{old}}\right\} \end{array}$$

where ${\hat{C}}_{m}$ is the specific membrane capacitance.

### 2.2 Simulations of afferent driven activity

AMPA and NMDA excitatory synaptic currents were placed at the distal dendrite compartment and modeled by the following equations:

$$I_{\text{AMPA}}=g_{\text{AMPA}}r_{\text{AMPA}}\left(V_{m}‐E_{\text{AMPA}}\right);\;g_{\text{AMPA}}=4\;\text{nS},E_{\text{AMPA}}=0\;\text{mV}$$

$$\begin{array}{cc} & I_{\text{NMDA}}=\frac{g_{\text{NMDA}}r_{\text{NMDA}}\left(V_{m}‐E_{\text{NMDA}}\right)}{1+\exp\left(‐0.062V_{m}\cdot \left[\text{Mg}\right]/3.57\right)};\\ & g_{\text{NMDA}}=15\;\text{nS},E_{\text{NMDA}}=0\;\text{mV},\left[\text{Mg}\right]=1.5\;\text{mM} \end{array}$$

$$\begin{array}{cc} & \frac{dr_{\text{AMPA}}}{dt}=\alpha_{\text{AMPA}}\left[\text{GLU}\right]\left(1‐r_{\text{AMPA}}\right)‐\beta_{\text{AMPA}}r_{\text{AMPA}};\\ & \alpha_{\text{AMPA}}=1.1{\left(\text{mM}\cdot \text{ms}\right)}^{‐1},\;\beta_{\text{AMPA}}=0.19\;{\text{ms}}^{‐1} \end{array}$$

$$\begin{array}{cc} & \frac{dr_{\text{NMDA}}}{dt}=\alpha_{\text{NMDA}}\left[\text{GLU}\right]\left(1‐r_{\text{NMDA}}\right)‐\beta_{\text{NMDA}}r_{\text{NMDA}};\\ & \alpha_{\text{NMDA}}=0.072{\left(\text{mM}\cdot \text{ms}\right)}^{‐1},\;\beta_{\text{NMDA}}=0.0066\;{\text{ms}}^{‐1} \end{array}$$

Glutamate release was modeled by the following step function

$$\left[\text{GLU}\right]=\left\{\begin{array}{l} 1\;\text{mM}\;t_{\text{on}}\leq t\leq t_{\text{on}}+1\;\text{ms}\\ 0\;\text{mM}\text{otherwise} \end{array}\right)\;$$

where [GLU] is the glutamate concentration at the synaptic cleft.

### 2.3 Numerical integration

To avoid numerical stability issues the derivates in the equations where approximated by backward finite differences

$$\frac{dV_{X}}{dt}\approx \frac{V_{X}^{\left(t\right)}‐V_{X}^{\left(t‐\Delta t\right)}}{\Delta t}$$

obtaining the following matrix equation

$$\begin{array}{c} \left(\begin{array}{ccc} \left[1+\left(G_{S}+g_{\text{SP}}\right)\Delta t/C_{S}\right] & ‐g_{\text{SP}}\Delta t/C_{S} & 0\\ ‐g_{\text{SP}}\Delta t/C_{P} & \left[1+\left(G_{P}+g_{\text{SP}}+g_{\text{PD}}\right)\Delta t/C_{P}\right] & ‐g_{\text{PD}}\Delta t/C_{P}\\ 0 & ‐g_{\text{PD}}\Delta t/C_{D} & \left[1+\left(G_{D}+g_{\text{PD}}\right)\Delta t/C_{D}\right] \end{array}\right)\left\{\begin{array}{c} V_{S}^{\left(t\right)}\\ V_{P}^{\left(t\right)}\\ V_{D}^{\left(t\right)} \end{array}\right\}\\ =\left(\begin{array}{c} V_{S}^{\left(t‐\Delta t\right)}+E_{S}\Delta t/C_{S}+I_{\text{CLAMP}}^{\left(t\right)}\\ V_{P}^{\left(t‐\Delta t\right)}+E_{P}\Delta t/C_{P}\\ V_{D}^{\left(t‐\Delta t\right)}+E_{D}\Delta t/C_{D} \end{array}\right) \end{array}$$

where $G_{S}=\sum_{X}g_{\text{X,S}}\left(V_{S},t\right),\;G_{P}=\sum_{X}g_{\text{X,P}}\left(V_{P},t\right),\;G_{D}=\sum_{X}g_{\text{X,D}}\left(V_{D},t\right)$.

As the above coefficient matrix is non‐singular the latter equation was solved by matrix inversion. The numerical iterations were programmed and run in Mathematica 10.4 software using a time step of 0.1 ms.

## 3 RESULTS

### 3.1 The model captures the characteristics features of DA cell electrophysiology

Figure 1 depicts the model behavior. The spike count for a simulation time length of 6 seconds is 13 (Figure 1b). Injecting a constant current of −35.1299 pA to the soma compartment clamps the membrane potential to −70 mV, while the membrane time constant relaxes to around 28.4 ms (Figure 1c). At this potential, the model's input resistance is near 278.7 MΩ (Figure 1d1,2). Placing a 20 nS NMDA conductance at the model's soma elicits a burst response similar to that recorded during dynamic clamp experiments in the midbrain slice preparation (Figure 1e) (Lobb et al., 2010, 2011). As in other DA neuron models (Komendantov et al., 2004; Kuznetsova et al., 2010), what drives the pacemaker activity is the L‐type Ca^++^ conductance with no contribution of the h‐current (Figure 1f,g). Although in mice *I*_h_ appears to contribute to the spontaneous activity (Okamoto et al., 2006), in the rat brain slice this remains controversial. Here we used an *I*_h_ voltage dependence and reversal potential similar to that of Hopf et al. (Hopf et al., 2007), where bath application of the h‐channel blocker ZD7288 did not alter intrinsic DA cell activity. Notice that in the model although *I*_h_ does not influence spontaneous spiking the h‐conductance response to membrane hyperpolarization is robust (Figure 1h, i1, and i2).

### 3.2 Effects of cell size alterations on intrinsic activity

A reduction in neuronal size implies a diminished cell surface area, which may result in a lower value of membrane conductance since now there is less membrane to insert ion channels. Cell capacitance (*C*_m_) will also be decreased as this parameter is directly proportional to the membrane surface area. All simulations in this work regarding the effects of size alterations are, therefore, executed by modifications in *C*_m_ similar to that observed in rodent models of drug addiction (see Materials and Methods) (Arencibia‐Albite et al., 2012; Mazei‐Robison et al., 2011).

In morphine‐exposed mice, a reduction in DA cell size, that shrinks the mean cell surface area by nearly 30%, is accompanied by a substantial reduction in the expression of various K^+^ conductances plus a significant increment in autonomous spiking (Mazei‐Robison et al., 2011). This suggests that in order to normalize the excitability of the affected cell it is necessary to return the magnitude of all distinct populations of ion channels to its pre‐morphine level. Or equivalent, a DA cell that diminishes its size but conserves the pre‐treatment number of all membrane ion channels should maintain an unaltered spontaneous activity. We next test the validity of this claim by reducing the capacitance of the model neuron in the presence of an unchanged conductance, that is, the peak magnitude of each conductance is kept constant in all compartments throughout all simulations.

**FIGURE 2:**
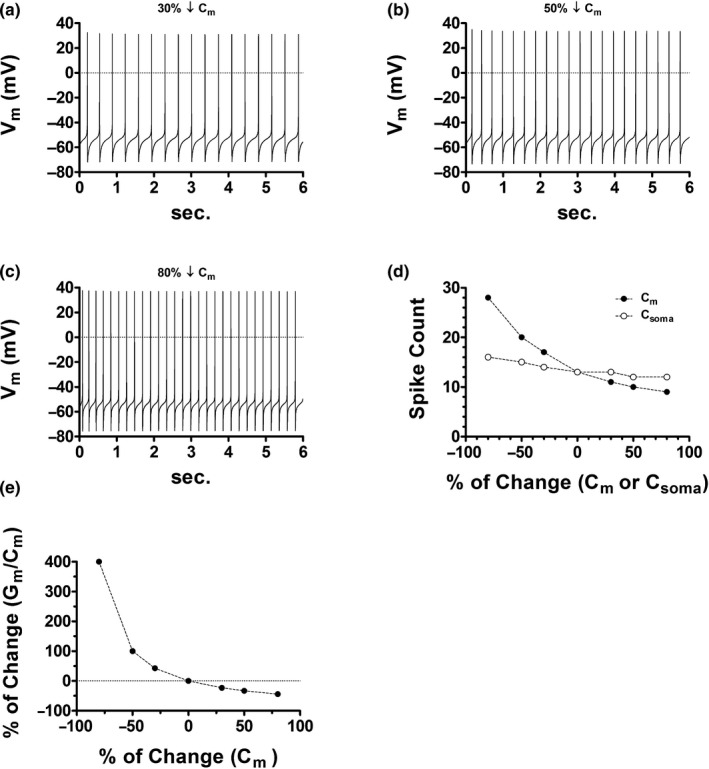
Effects of DA cell size attenuation in the presence of a fixed number of membrane ion channels. (a) A *C*_m_ reduction of 30% elevates cell firing by ~31% (from 2.16 to 2.83 Hz) and increases the action potential amplitude (APA) by 5.98 mV (from 98.46 to 104.44 mV). (b) A *C*_m_ reduction of 50% elevates cell firing by ~54% (from 2.16 to 3.33 Hz) and increases the APA by 9.86 mV (from 98.46 to 108.32 mV). (c) A *C*_m_ reduction of 80% elevates cell firing by ~115% (from 2.16 to 4.66 Hz) and increases the APA by 14.98 mV (from 98.46 to 113.44 mV). (d) Scatter plot that summarizes the effects of cell size alterations in the presence of a fixed number of membrane ion channels. (e) Scatter plot showing that a decline in *C*_m_, under a fixed number of membrane ion channels, results in a substantial elevation of the densities of each channel type. For example, a 30% drop in capacitance elevates densities by about 43%.

Figure 1b shows the control condition where the model generates 13 spikes in 6 seconds. In Figure 2a, a 30% reduction in cell capacitance increases the spike count to 17. Likewise, a reduction of 50% (Figure 2b) and (Figure 2c) 80% results in spike counts of 20 and 28, respectively. Figure 2d summarizes simulations and shows that a reduction in whole‐cell capacitance, in the presence of a non‐changing number of ion channels, increases the intrinsic activity (black dots). The reduction of the somatic capacitance alone, in contrast, evoked non‐significant increments in spontaneous firing (white dots). These simulations illustrate, consequently, that conserving a constant number of ion channels does not prevent the increase in spiking frequency that follows from a reduction in cell size.

Figure 2e illustrates that a reduced cell capacitance in the presence of a constant peak conductance level implies a substantial relative increment in ion channel density (i.e., number of ion channels per unit of membrane surface area). This signifies that, as a homeostatic response, the conservation of a constant number of ion channels represents a futile adaptation to cell size diminution since the metabolic cost that underlies the elevated density does not halt the increase in spiking frequency. The next section shows how the channel density scaling, which results from a reduced cell size, affects intrinsic activity.

### 3.3 Effects of ion channel density scaling on intrinsic activity

In the next simulations as the cell capacitance is decreased by 30% the peak magnitude of all membrane conductances (denoted by *G*_m_) will be unchanged, increased, or decreased relative to its pre‐reduction magnitude. These computational experiments illustrate how the intrinsic activity responds to changes in the net current density time course that are induced by alterations in ion channel densities. In these numerical recreations when *G*_m_ is altered by a fixed proportion, the peak magnitude of each conductance in all compartments has been scaled by the same proportion. We proceeded in this manner for two reasons. First, it is unlikely that a change in intrinsic activity, secondary to cell size reduction, is the result of alterations in exactly a single conductance type. Second, in order for the DA cell to conserve its pacemaker activity, after size diminution, the relative change in the magnitude of outward currents cannot be much greater or much smaller than that of inward currents since both situations may lead to the abolition of spontaneous spiking. Therefore, in order for the intrinsic activity to survive an event of substantial size reduction, the variability among the different relative changes ascribed to each conductance has to be small. A reasonable approximation to such a case is to assume uniform scaling among the distinct membrane conductances. This approach, as shown next, clearly illustrates how intrinsic activity adjusts to modifications in ion channel density.

**FIGURE 3:**
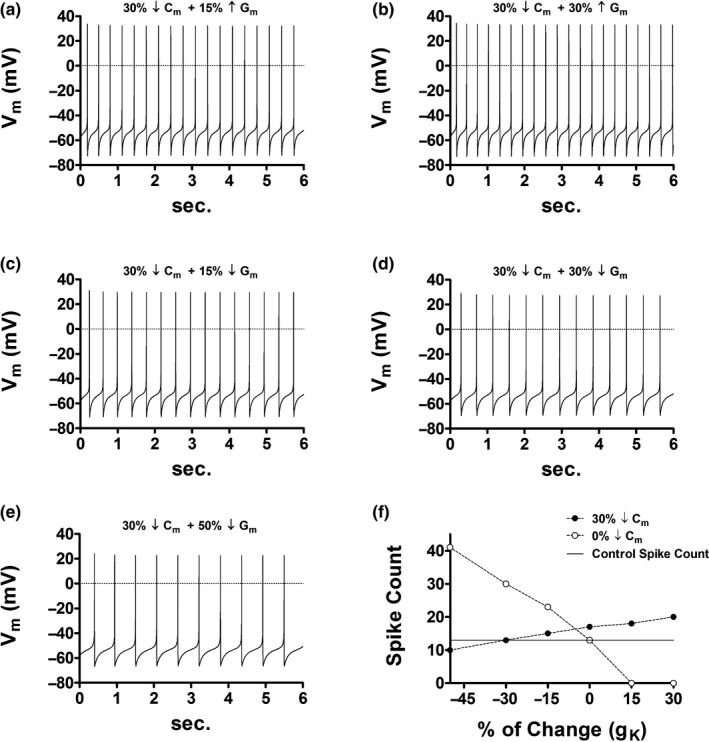
Effects of channel density scaling secondary to DA cell size reduction. (a) A reduction of 30% in *C*_m_ with a 15% increase in *G*_m_ elevates the channel density of each conductance by ~64%, in contrast, to control (Figure 1b). In this case, the spike count has augmented by five spikes (from 13 to 18; 2.16 Hz → 3 Hz). Notice here that *g*_K_ has augmented by 15% yet cell firing is enhanced. (b) A reduction of 30% in *C*_m_ with a 30% increase in *G*_m_ elevates the channel density of each conductance by ~86%, in contrast, to control (Figure 1b). In this case, the spike count has augmented by seven spikes (from 13 to 20; 2.16 Hz → 3.33 Hz). Notice here that *g*_K_ has raised by 30% yet cell firing is enhanced. (c) A reduction of 30% in *C*_m_ with a 15% decrease in *G*_m_ elevates the channel density of each conductance by ~21%, in contrast, to control (Figure 1b). In this case, the spike count has augmented by two spikes (from 13 to 15; 2.16 Hz → 2.5 Hz). When contrasted to Figure 2a, the 15% declined in *g*_K_ correlates with cell excitation dampening and not with enhance cell firing. (d) A reduction of 30% in *C*_m_ with a 30% decrease in *G*_m_ conserves the channel density of each conductance. In this case, the spike count and the membrane voltage trace remain identical to control (Figure 1b). When contrasted to parts a, b, and c, the 30% declined in *g*_K_ correlates with the preservation of cell excitation and not with enhance cell firing. (e) A reduction of 30% in *C*_m_ with a 50% decrease in *G*_m_ decreases the channel density of each conductance by ~29%, in contrast, to control (Figure 1b). In this case, the spike count has declined by three spikes (from 13 to 10; 2.16 Hz → 1.66 Hz). When contrasted to parts a, b, c, and d, the 50% declined in *g*_K_ correlates with cell excitation dampening and not with enhance cell firing. (f) Scatter plot that summarizes the above simulations (black dots). The horizontal axis has been label as *g*_K_, and not *G*_m_, to emphasize that a decrease in *g*_K_, after size reduction, correlates with cell excitation dampening and not with enhance cell firing. A decrease in *g*_K_ increases cell excitation only when all other cell properties are held constant (white dots).

Panels a and b in Figure 3 illustrate that when a drop in cell capacitance is followed by a rise in *G*_m_ the spike count also increases. Panels c–e show, in contrast, that a decline in *G*_m_ rescues the intrinsic activity only if the relative change in *G*_m_ matches that in *C*_m_. Consequently, is possible to conserve the spiking frequency, after cell size attenuation, by just preserving the channel density for each conductance present in the membrane.

Figure 3 also challenges a widespread principle in neuroscience: “a drop in K^+^ conductance augments cell excitation.” Panel f summarizes all simulations in Figure 3 and shows, for example, that under a reduced cell size a decline in the net K^+^ conductance (*g*_K_) tends to depress intrinsic activity and not to enhance it as suggested by this empirical norm. As cell size diminishes there is less membrane area to insert ion channels and hence the net conductance for each permeable ion is likely to be decreased. As a result, when other membrane elements are changing is not possible to predict alterations in cell excitation by simply inspecting if *g*_K_ has rise, not change or decay. In effect, in Figure 3d *g*_K_ has decreased by 30% which by itself it is expected to increase cell excitation, yet as seen in the simulation the intrinsic activity remains unchanged relative to the control case. Thus, a reduction in *g*_K_ correlates with increase excitation only when all other biophysical properties are held constant (white dots in Figure 3f).

### 3.4 The ether‐a‐go‐go related gen K^+^ current may prevent the increased DA cell firing secondary to size reduction

A decline in cell size diminishes cell surface area leading to alterations in channel densities. If the tendency, subsequent to size attenuation, is to raise the membrane concentration of all ion channels then the autonomous firing frequency is likely to be elevated (see Figure 3a,b). Is possible, as previously discussed, to rescue the normal intrinsic activity by conserving the original ratios of the number of channels per unit of area (see Figure 3d). This section reveals, nonetheless, a remarkable and unexpected biophysical mechanism that effectively buffers the increased DA cell excitation secondary to size reduction. Such a mechanism does not require the preservation of the channel density for each conductance.

DA neurons from the rat express the ether‐a‐go‐go related gen K^+^ (ERG) current termed *I*_ERG_ (Canavier et al., 2007; Ji et al., 2012). In these cells, the selective blockade of ERG channels increased the frequency of spontaneous activity as well as the firing response to current injection and also accelerates the entry into depolarization block as evoked by dynamic clamp bursting (Ji et al., 2012). These properties, as explained next, result from the *I*_ERG_ activation kinetics and voltage dependence.

ERG channels activate slowly with depolarization but, as the amplitude of stimulus increases, inactivation occurs almost immediately after channel opening (Ficker et al., 2001). Therefore, during the upward phase of the somatic spike, a substantial proportion of ERG channels have transitioned into the inactive state. During the downward phase, as inactive ERG channels return to the close state, they must pass first through the open conformation. The acquisition of the open state is nearly instantaneous; however, open ERG channels recover the close state at a much slower rate resulting in a significant outward current between consecutive action potentials. Consequently, ERG channels limit cell firing by providing a robust interspike outward current that dampens the intensity of depolarizing currents.

**FIGURE 4:**
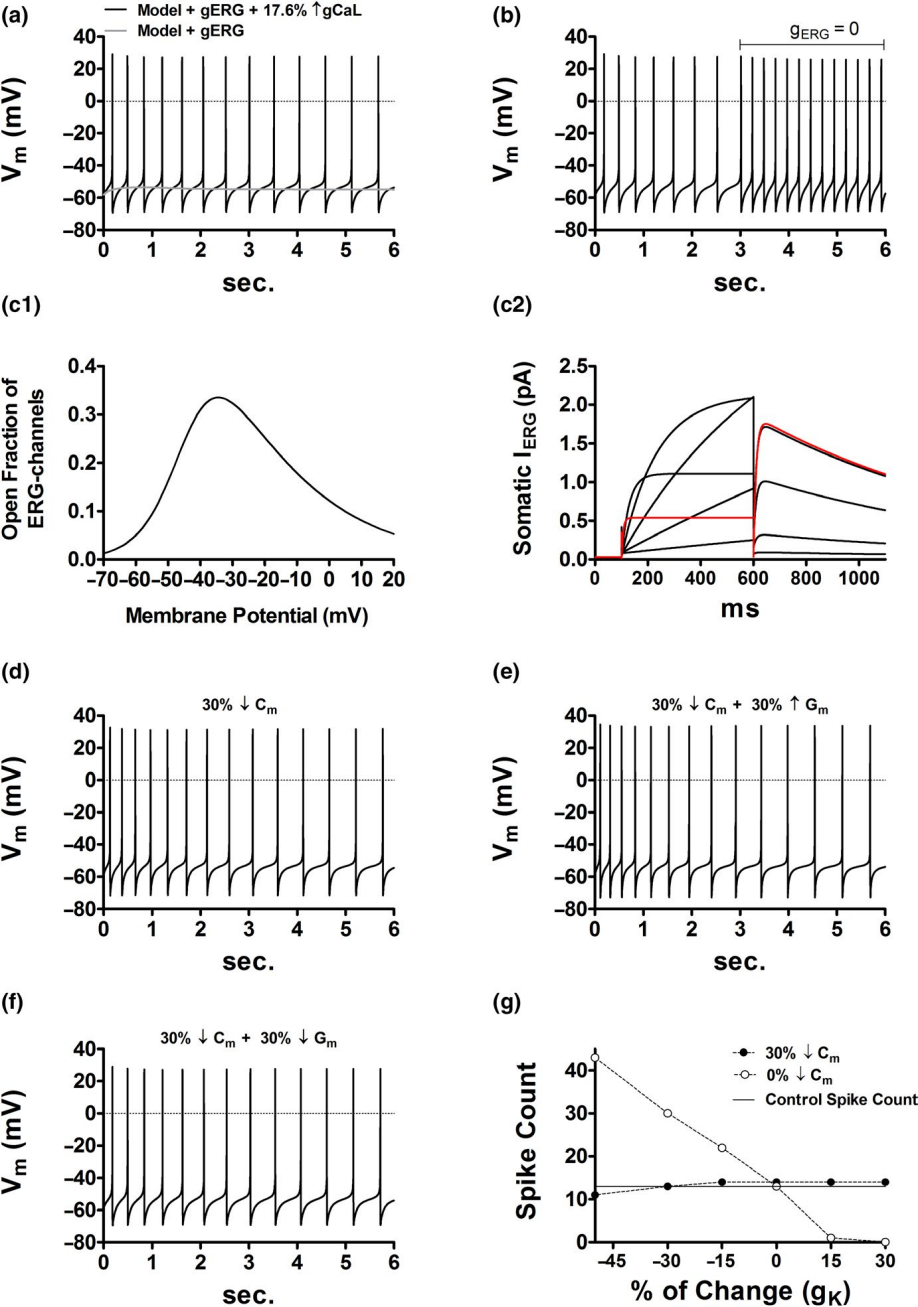
The ether‐a‐go‐go related gen K^+^ (ERG) current may prevent the increased DA cell firing secondary to size reduction. (a) The initial placement of the ERG conductance in the DA neuron model breaks the balance between inward and outward currents abolishing intrinsic activity (gray trace). After placing the ERG conductance in all compartments, the control firing frequency is recovered by increasing the non‐inactivating L‐type Ca^++^ conductance (*g*_CaL_). (b) The blockade of *I*_ERG_ doubles the firing frequency (from 2.16 to 4.33 Hz); the ERG conductance (*g*_ERG_) was set to zero in all compartments. (c1) ERG channel activation curve. (c2) *I–V* curve of the somatic *I*_ERG_. Holding potential was −70 mV. Step commands were from −50 to 50 mV in 20 mV increments. The red trace is the current response to the 50 mV step. (d) In the absence of *I*_ERG_, a 30% reduction in *C*_m_, with no change in *G*_m_, increases the spike count by four spikes (from 13 to 17; 2.16 → 2.83 Hz, see Figure 2a). In the presence of *I*_ERG_, however, the spike count increases by one spike (from 13 to 14; 2.16 → 2.33 Hz). (e) In the absence of *I*_ERG_, a 30% reduction in *C*_m_, with a 30% increase in *G*_m_, increases the spike count by seven spikes (from 13 to 20; 2.16 → 3.33 Hz, see Figure 3b). In the presence of *I*_ERG_, however, the spike count increases by one spike (from 13 to 14; 2.16 → 2.33 Hz). (f) A reduction of 30% in *C*_m_ together with a 30% decrease in *G*_m_ conserves the spike count. In this case, the membrane voltage trace remains identical to control (Figure 4a). (g) Scatter plot that summarizes the simulations in the presence of *I*_ERG_ (black dots). The horizontal axis has been label as *g*_K_, and not *G*_m_, to emphasize that when other biophysical attributes are changing is not possible predict the adjustments in cell firing by just measuring the expression level of *g*_K_; *g*_K_ is inversely related to cell excitation only when all other membrane properties are held constant (white dots).

Figure 4a shows the incorporation of the ERG conductance into the computational model. In accordance with experimental data (Ji et al., 2012), the inhibition *I*_ERG_ in the model increases intrinsic activity (Figure 4b). The activation curve of the ERG channel demonstrates that at voltages close to the peak of the action potential the fraction of open channels is non‐significant (Figure 4c1). The *I*_ERG_ IV curve shows that ERG channel inactivation increases as the amplitude of the command potential increases (Figure 4c2). Yet, as the repolarization amplitude augments, tail currents become larger and last longer (Figure 4c2). Consequently, if during cell size reduction the ERG channel density increases the rise in firing frequency could be significantly dampened. This claim is reasonable for two reasons. First, a reduced cell size increases the amplitude of action potentials (see Figure 2) which, in turn, augments the fraction of inactive ERG channels. Second, on repolarization, a large inactive fraction leads to a large open fraction that when combined with an increased channel density enhances *I*_ERG_ density. This may slow down the elevated depolarizing drive that emerges with diminished cell morphology.

**FIGURE 5:**
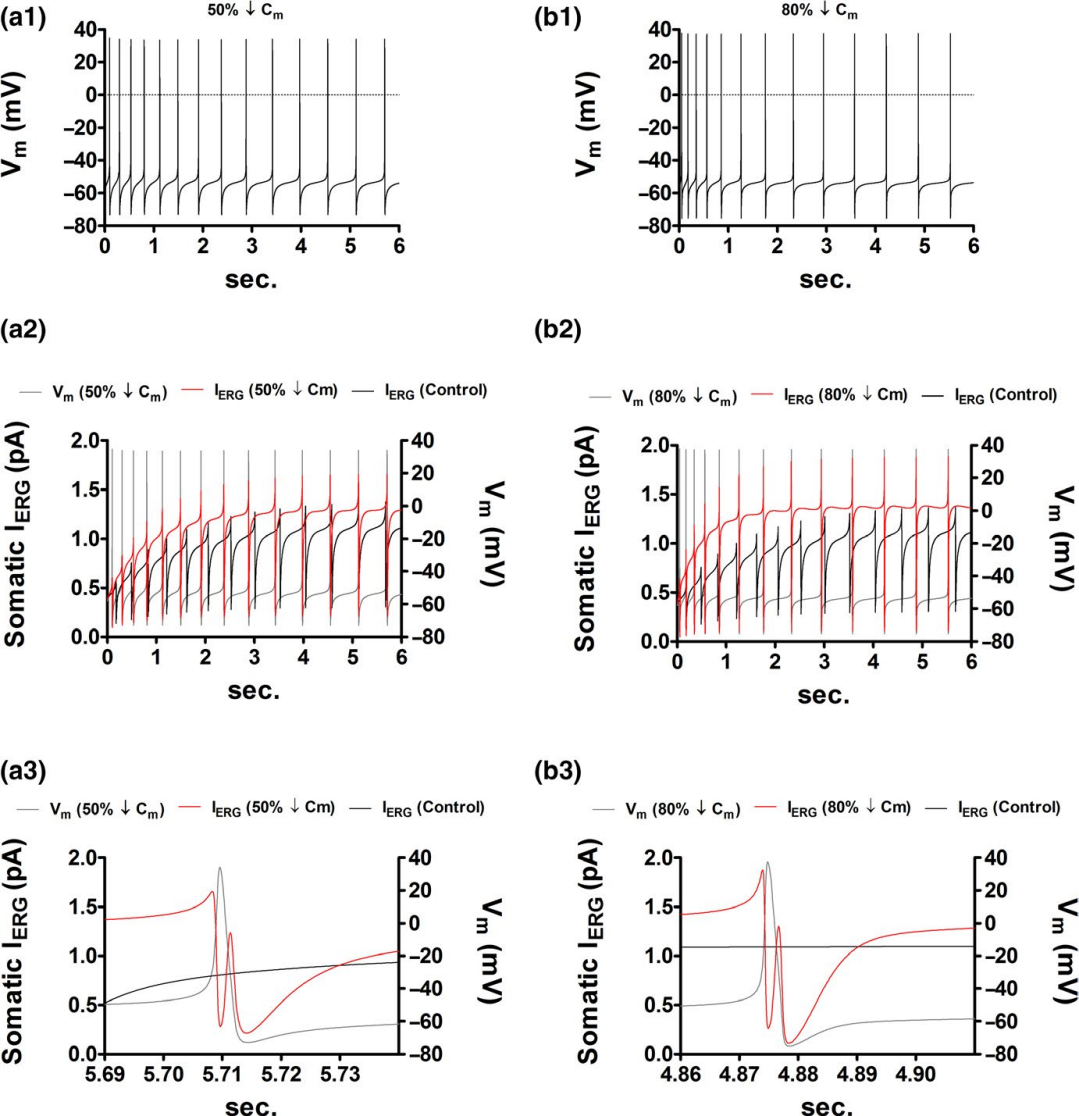
I_ERG_ amplitude increases in response to cell size reduction. (a1) In the absence of *I*_ERG_, a reduction of 50% in *C*_m_, with no change in *G*_m_, increases the spike count by seven spikes (from 13 to 20; 2.16 → 3.33 Hz, see Figure 2b). In the presence of *I*_ERG_, however, the spike count increases by one spike (from 13 to 14; 2.16 → 2.33 Hz). The APA was increased by 9.64 mV (from 98.46 to 108.1 mV). (a2) The increment in APA measured in a1 increases *I*_ERG_ activation (black trace vs. red trace). The somatic *I*_ERG_ mean value was augmented by ~22% (from 0.91 to 1.11 pA). Such enhanced amplitude was able to effectively dampen the expected raise in intrinsic firing after a 50% *C*_m_ reduction. I_ERG_ mean value is defined as ${\bar{I}}_{\text{ERG}}=\frac{1}{6}\int_{0}^{6}I_{\text{ERG}}dt$. The *V*_m_ gray trace is identical to the *V*_m_ trace in a1. (a3) The graph expands the content in a2 in the shown time interval. (b1) In the absence of *I*_ERG_, a reduction of 80% in *C*_m_, with no change in *G*_m_, increases the spike count by 15 spikes (from 13 to 28; 2.16 → 4.66 Hz, see Figure 2c). In the presence of *I*_ERG_, however, the spike count remains constant. The APA was increased by 14.93 mV (from 98.46 to 113.39 mV). (b2) The APA increment measured in C1 increases the *I*_ERG_ mean value by ~37% (from 0.91 to 1.25 pA). This elevated amplitude was sufficient to prevent the expected raise in intrinsic firing after an 80% *C*_m_ reduction. The *V*_m_ gray trace is identical to the *V*_m_ trace in b1. (b3) The graph expands the content in b2 in the shown time interval.

We next address the latter hypothesis by repeating simulations in Figure 3 but now in the presence of *I*_ERG_. Figure 4a is the control condition, and as expected, a reduction of 30% in cell capacitance, while *G*_m_ remains constant (Figure 4d) or increased up to 30% (Figure 4e), augments the spike count by just one spike. Notice also that the action potential waveform and number remain identical to control only when the relative drop in *G*_m_ is equal to that in cell capacitance (Figure 4f). Black dots in Figure 4g summarize simulations and suggest that in the presence of *I*_ERG_ cell size attenuation is unable to evoke a substantial elevation in the intrinsic firing. Figure 4g also emphasizes, once again, that when other membrane elements are changing is not possible to predict alterations in cell excitation by just examining the expression level of *g*_K_. A reduction in *g*_K_ increases cell excitation only when all other membrane properties are clamped (white dots in Figure 4g). Figure 5 substantiates further the addressed hypothesis as it illustrates instances where *I*_ERG_ amplitude increases with decreasing cell size.

### 3.5 Effects of cell size attenuation on afferent‐driven activity

The DA neuron model is next current‐clamped at −55 mV and excited at the distal dendrite. Intermittent synaptic excitation onto the latter compartment is simulated by a train of ten 1 mM glutamate pulses (duration: 1 ms) at a frequency of 33⅓ Hz. Such stimulus evokes the transient activation of AMPA and NMDA ligand‐gated channels resulting in the somatic summation of 10 excitatory post‐synaptic potentials (EPSPs). The maximal values of these dendritic conductances are fixed in all simulations.

**FIGURE 6:**
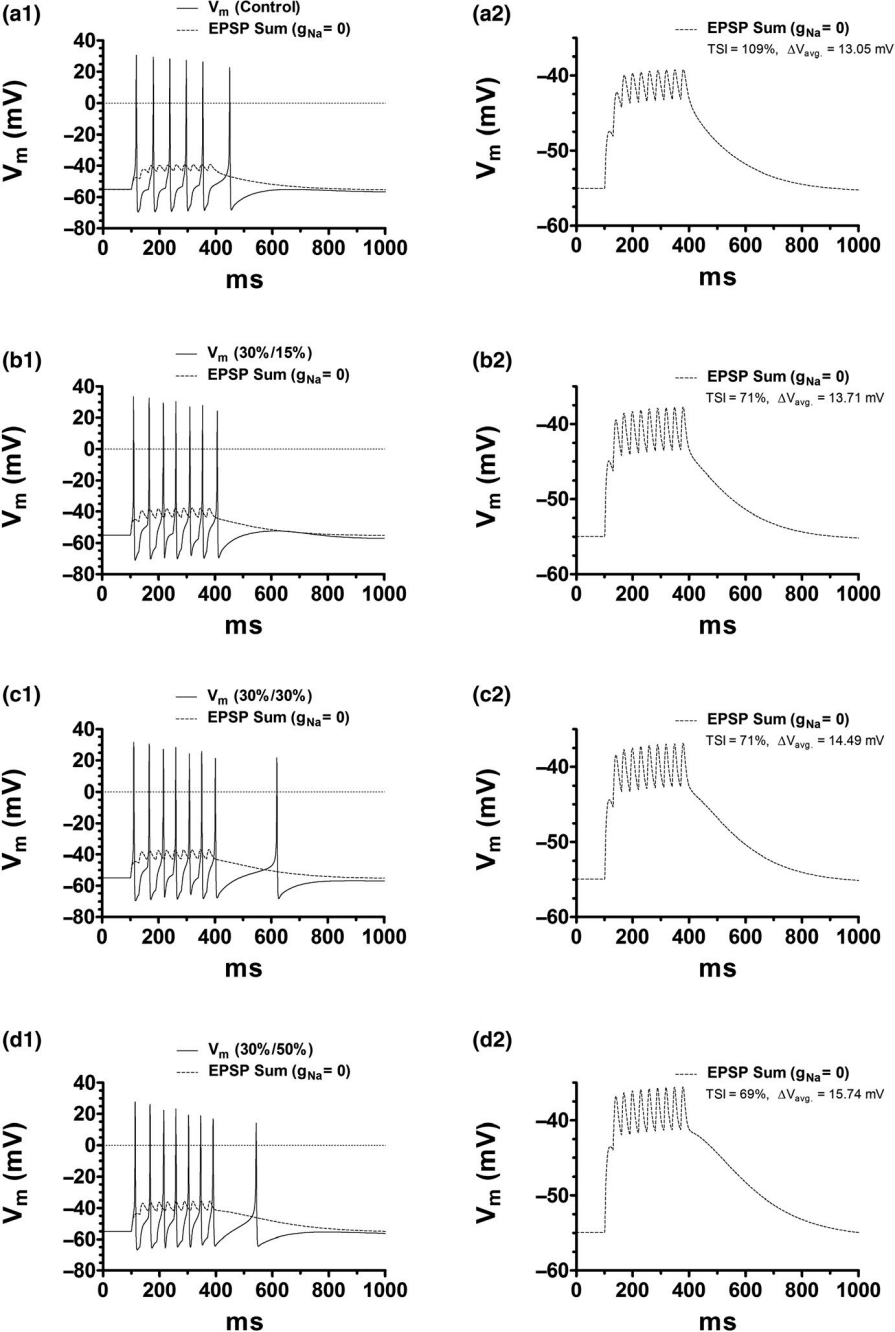
DA cell size reduction results in a paradoxical enhancement of the afferent‐evoked burst signal. AMPA (4 nS) and NMDA (15 nS) channels were placed in the distal dendrite. This compartment was then excited by a train of 10 glutamate squared pulses. Simulations in this figure include the ERG conductance in all compartments as no substantial differences were observed in its absence (data not shown). (a1) Control burst signal at the soma compartment. The EPSP summation that elicits this response is exposed by setting *g*_Na_ = 0 in all compartments (dashed curve). *C*_m_ and *G*_m_ were held fixed. (b1) Burst signal after *C*_m_ and *G*_m_ are decreased by 30% and 15%, respectively. (c1) Burst signal after *C*_m_ and *G*_m_ are both decreased by 30%. (d1) Burst signal after *C*_m_ and *G*_m_ are decreased by 30% and 50%, respectively. a2, b2, c2, d2. Zoom in on the temporal summation recording in part 1. In c2, b2, and d2, the TSI has experienced a substantial depression relative to that of the control recording (a2), yet the average membrane depolarization has increased. Such behavior represents a paradoxical response according to the current view of the TSI. Notice that, in all figures relative to a1, the elevated average membrane depolarization is what underlies the reduced inter‐spike interval and elevated spike count.

Figure 6a1 illustrates the control case where the train stimulus triggers a burst of 6 spikes. The EPSPs integration that underlies this response can be observed by setting the Na^+^ conductance (*g*_Na_) to zero in all compartments (Figure 6a1,a2). Here the temporal summation index (TSI) is given by the relative change in the last EPSP with respect to the first, that is, $\text{TSI}=\left({\text{EPSP}}_{\text{last}}/{\text{EPSP}}_{\text{first}}‐1\right)\times 100$. Our previous work (Arencibia‐Albite et al., 2017) has shown that, under cell size attenuation, the TSI may be a poor indicator of the quality of synaptic integration. We proposed that to better assess the level of temporal summation, the TSI has to be accompanied by the computation of the average membrane depolarization (Δ*V*_avg_); defined as

$$\Delta V_{\text{avg}}=\frac{1}{T}\int_{0}^{T}\Delta V_{m}dt$$

where Δ*V*_m_ is the change in membrane potential with respect to the holding voltage and *T* represents the time span between the peak values of the first and last EPSP. As TSI increases Δ*V*_avg_ is expected to increases since these measurements are usually positively correlated (Angelo et al., 2007; Brager & Johnston, 2007; Carr et al., 2007; Lewis et al., 2011). Therefore, if cell size reduction improves afferent‐driven activity both measurements should augment after size attenuation.

Figure 6b1,b2 illustrate the model response when *C*_m_ and *G*_m_ are decreased by 30% and 15%, respectively. Notice that the enhanced burst signal results from the augmented Δ*V*_avg_ yet TSI has diminished by 38%. Likewise, decreasing *G_m_* by 30% (Figure 6c1) or 50% (Figure 6d1), in the presence of a 30% drop in *C*_m_, further increases the spike count (Figure 6c1,d1). The TSIs in these cases are also reduced, while the respective Δ*V*_avg_ values are increased (Figure 6c2,d2). Altogether it suggests that diminished cell size is associated with a reduced TSI that paradoxically correlates with an elevated Δ*V*_avg_ and hence an improved afferent‐driven activity; similar to what we have shown in an animal model of chronic cocaine administration (Arencibia‐Albite et al., 2017).

## 4 DISCUSSION

### 4.1 The positive correlation, between firing frequency and input resistance, only holds when the cell size is fixed

A reduced DA cell size, induced by morphine exposure or by Clock gene mutation, correlates with an increased autonomous firing (Coque et al., 2011; Mazei‐Robison et al., 2011; Mcclung et al., 2005). This finding is believed to be primarily the result of a reduction in K^+^ channel expression (Mazei‐Robison et al., 2011); which, as argued by other theoretical studies, increases the intrinsic spiking rate as a consequence of the rise in the cell's input resistance (Enrico et al., 2016; Sengupta et al., 2013). If this analysis is correct then the appropriate homeostatic response that restores spontaneous activity is the conservation of the pretreatment population size for each ion channel type; which signifies an elevated channel density for each distinct membrane conductance. Although such adaptation preserves the input resistance it did not prevent the raise in cell firing as shown here. Our computational experiments illustrate, in contrast, that the response that conserves the pretreatment activity is the preservation of the channel density of each conductance, which elevates the input resistance as cell size diminishes. Consequently, is likely that the down‐regulation of *g*_K_, under cell size attenuation, dampens the raise in intrinsic activity by decreasing the K^+^ channels density. This furthers implies that the proposed positive correlation between firing frequency and input resistance, only holds when the cell size or capacitance is kept fixed; otherwise, is not possible to infer the change in cell firing by only measuring the change in input resistance.

### 4.2 The conservation of ion channels densities, after cell size reduction, preserves the rate and pattern of spontaneous firing

The Appendix section explains in detail how the conservation of the channel density of each conductance preserves the rate and pattern of spontaneous firing. The given analytical argument also elucidates why a constant number of ion channels does not halt the rise in intrinsic firing associated with a decrease in cell morphology. The biophysical interpretation of the argument is remarkably simple: if the number of channels is kept constant then, under cell size reduction, the inward and outward currents will be elevated per unit of membrane surface area and thus the time required to charge and discharge the now smaller cell membrane will be substantially diminished. Such a process is very similar to the problem of how many times, during a fixed time period, a water tank can be filled and emptied: If the inflow and outflow rates are kept constant then the number of fill‐empty cycles will increase when the tank size is reduced. This is in essence what explains the augmented firing frequency as cell size decreases, while the number of ion channels remains clamped.

### 4.3 Somatic size reduction has minimal impact on spontaneous cell activity

Enrico et al. (Enrico et al., 2016) had implemented a realistic DA neuron model of 28 compartments (soma, 11 proximal dendrites and 16 distal dendrites) to analyze the biophysical effects of opiates withdrawal. In their work, size reduction was restricted to the somatic compartment since as explained by these authors morphine effects on the structure of the dendrites are not clear. Modifications in cell firing after somatic shrinkage were, however, non‐substantial. In agreement with this finding, cell spiking in our model also experienced minor changes when size contraction was limited to only somatic dimensions. It seems, therefore, that in order for a given morphological alteration to impact cell activity it needs to affect a significant proportion of the whole‐cell surface area; if not the effects appear to be inconsequential. As a result, the present theoretical study suggests that in order for morphine exposure or Clock gene mutation to evoked a significant raise in the spontaneous activity it has to also reduced a significant proportion of the dendrites surface area. Further experimentation is required to test such a prediction.

### 4.4 The presence of the ERG current may limit the rise in spontaneous activity after cell size reduction

In rats repeated cocaine administration results in a substantial decline in the whole‐cell capacitance of DA cells (Arencibia‐Albite et al., 2012, 2017). In contrast to mice, the spontaneous activity remains apparently unaltered after capacitance reduction (Arencibia‐Albite et al., 2012). A possible explanation for this discrepancy could be the presence of the ERG current. In rats the ERG conductance is highly expressed and is known to decrease the level of intrinsic cell firing by providing a strong interspike outward current (Ji et al., 2012). The outstanding aspect of this K^+^ current is that the open fraction of ERG channels increases as the spike's repolarization rate rises (see Figure 4c2). Thus, is possible, that the smaller capacitance that elicits a faster spike is also leading to a level of channel recruitment that exceeds the pre‐reduction open number; even if the ERG channels population has dropped after size attenuation. In agreement with this hypothesis, the addition of the ERG channel into the DA cell model was able to effectively buffer the rise in cell firing activity after size downscaling. The geometry of the discharge pattern was, however, visually distinct to that of the control simulation; the spiking pattern remains identical to the control state only when the channel density of each conductance was clamped (Figure 4f). Consequently, to rescue the spike waveform appearance, after size reduction, it is necessary to preserve the temporal interrelations among all distinct ionic currents; it cannot be achieved by changing the expression level of a single conductance in the isolation of the others.

### 4.5 Cell size attenuation correlates with an unusual improvement in synaptic integration

Synaptic summation in the model was paradoxically elevated after size contraction since it consisted of a significantly reduced summation indexed but a substantially increased depolarization. This numerical finding agrees with our previous experimental data that show that capacitance reduction improves the temporal summation of synaptic inputs in exactly the same manner as the simulations in this study (Arencibia‐Albite et al., 2017). This potentiated sub‐threshold activity was also associated with the enhancement of the burst signal in response to a fixed train of synaptic currents; a result consistent with data in mice showing that DA cell size reduction increases burst spiking (Coque et al., 2011; Mazei‐Robison et al., 2011; Mcclung et al., 2005). These observations suggest, overall, that the present model detail is adequate since its behavior is in close agreement with relevant published data.

## 5 CONCLUSION

**FIGURE 7:**
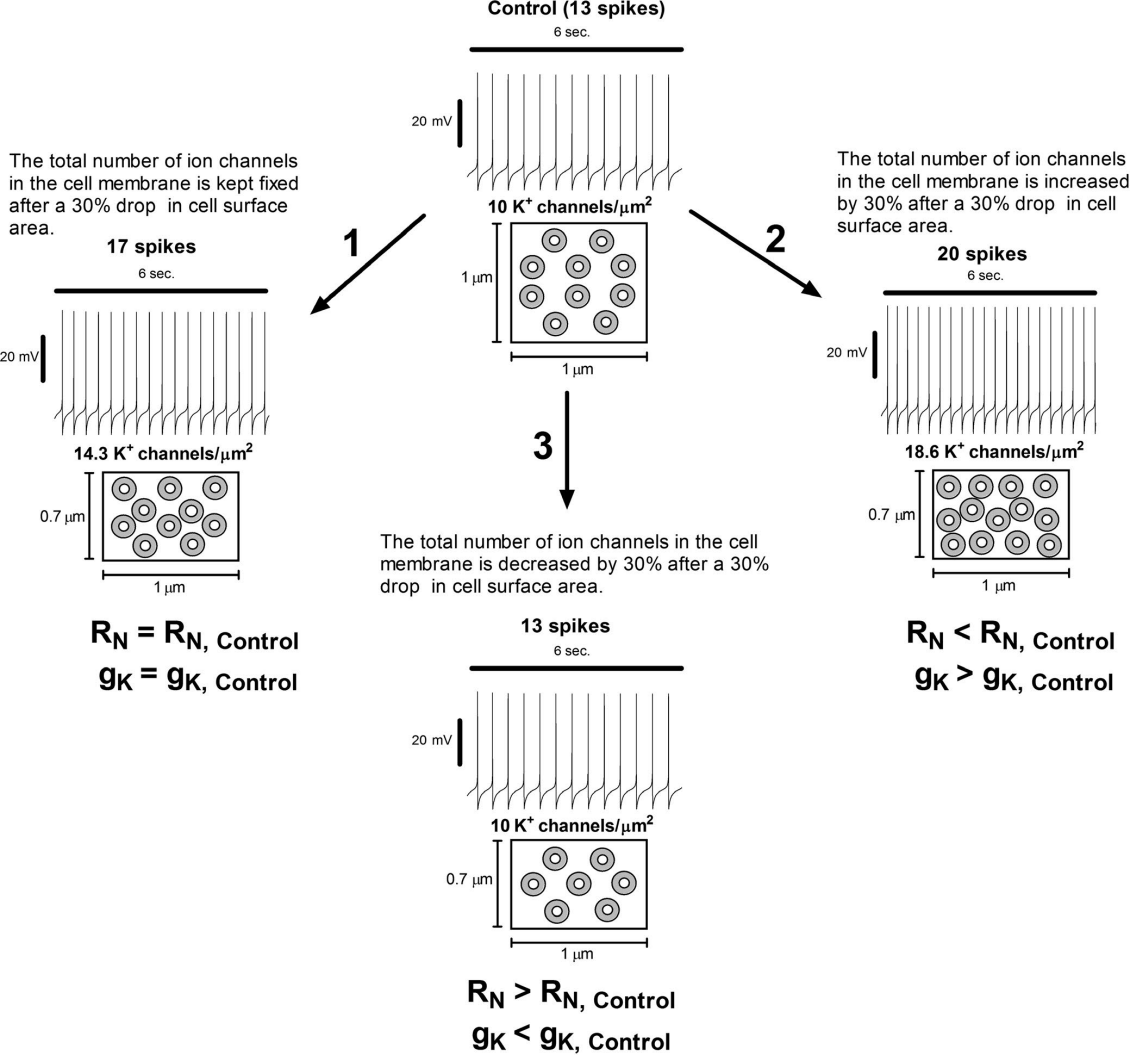
A hypothesis that experimental evidence seems to validate may be shown to be erroneous by numerical simulation. Chronic morphine exposure results in smaller midbrain dopamine neurons that express an elevated spontaneous firing which as argues by qualitative reasoning is simply a consequence of the rise in the cell's input resistance (*R*_N_) after size reduction. Motivated by this apparently reasonable hypothesis, experimental studies have shown that concomitant to this increased excitation *g*_K_ expression is substantially diminished and thus it is concluded that such finding is at least one of the main factors that elevate intrinsic activity. Computational analysis suggests, however, that we should rethink this conclusion even if the aforementioned evidence indicates otherwise. If this conclusion is correct then the appropriate homeostatic response that restores spontaneous activity should be the conservation of the pre‐exposure population size for each ion channel type; which elevates the number of channels per unit of membrane area for each distinct membrane conductance (see boxes below activity patterns in 1 and 2). The numerical recreation in 1 shows, nonetheless, that although this adaptation preserves *R*_N_ and *g*_K_ it did not prevent the elevation in cell firing. Moreover, in 2 *R*_N_ has dropped, while *g*_K_ has increased yet the cell spiking is not decelerated but further augmented. Surprisingly, and against the predominant descriptive reasoning that dominates the electrophysiological literature, 3 illustrates that the firing rate and spike waveform are insensitive to changes in cell size as long as the number of channels per unit of membrane area is preserved. Altogether, it signifies that the intrinsic firing pattern is only determined by the ion channel density of each conductance and not by the absolute magnitude of *R*_N_ or *g*_K_. Notice that such counterintuitive observation emerges as a consequence of computational analysis and not as a result of real experimentation. Consequently, a hypothesis that experimental evidence seems to validate may be shown to be erroneous by numerical simulation.

Simulations in this study confirm that the experimentally measured inverse relationship between *g*_K_ and the rate of intrinsic activity is only valid when all other biophysical properties remain fairly unaltered or fixed. Reductions in *g*_K_ that happen in parallel to cell size contraction tend to depress the augmentation in spontaneous activity. Computational experiments illustrate that the appropriate homeostatic response that conserves the pretreatment intrinsic spiking pattern and the rate is the conservation of the channel density of each conductance. This result is not a numerical artifact as it was analytically demonstrated. The presence of the K^+^ ERG conductance is also effective in dampening the rise in the autonomous firing as ERG channel activation increases as the spike depolarization rate increases; however, the spiking pattern remains clearly distinct from that of the control condition. Additionally, with cell size reduction, the standard measurement of synaptic summation appears depress yet the afferent‐driven burst activity is improved due to the enhanced depolarization secondary to the diminished cell capacitance. On the whole, this work demonstrates that experimentation in the absence of mathematical models may lead to the erroneous interpretation of the counterintuitive aspects of empirical data (Figure 7).

## Disclosure

No conflicts of interest, financial or otherwise, are declared by the authors.

## How a diminished cell size, with no change in the number of ion channels, augments spontaneous activity?

An autonomous spiking cell is analogous to a capacitor that is periodically charged and discharged. During each spontaneous spike, for instance, the cell membrane is first charged during the action potential upward phase and then discharged during the downward phase. The rate (i.e., d*V*_m_/d*t*) of such a process is independent of cell size and is ultimately determined by ion channels densities. To appreciate why, imagine, a 50 pF membrane with only *N* = 10,000 leak channels each having a unitary conductance γ = 1 pS (input conductance = *G*_m_ = *N*γ = 10,000 pS) and a reversal potential *E* = −60 mV. If this membrane is current‐clamped to *V*_0_ = −20 mV and then release, we know from electricity theory (Jack et al., 1975), that it would take about five time constants (5τ = 5*C*_m_/*G*_m_ = 5(50 pF/10,000 pS) = 5(5 ms) = 25 ms) to reach the resting potential *E*; a result that is independent of the value of *V*_0_. Notice also that τ is a function of the leak channels density ρ = N/Sa since

$$\tau =\frac{C_{m}}{G_{m}}=\frac{{\hat{C}}_{m}\cdot Sa}{N\cdot \gamma }=\frac{{\hat{C}}_{m}}{\left(\frac{N}{\text{Sa}}\right)\cdot \gamma }=\frac{{\hat{C}}_{m}}{\rho \cdot \gamma }$$

Thus, if *C*_m_ is decreased by 30%, while the number of leak channels (ch) is kept constant then ρ increases by 43%

$$\begin{array}{cc} & \rho_{\text{old}}=\frac{N}{{\text{Sa}}_{\text{old}}}=\frac{N}{C_{m}/{\hat{C}}_{m}}=\frac{10,000\;\text{ch}}{5,000\;{\mu m}^{2}}=2\;\text{ch}/{\mu m}^{2}\\ & \to \rho_{\text{new}}=\frac{10,000\;\text{ch}}{3,500\;{\mu m}^{2}}\approx 2.86\;\text{ch}/{\mu m}^{2} \end{array}$$

causing *τ* to decrease by 1.5 ms

$$\tau_{\text{new}}=\frac{{\hat{C}}_{m}}{\rho_{\text{new}}\cdot \gamma }=\frac{1\;\text{pF}/100\;{\mu m}^{2}}{\left(2.86\;\text{ch}/{\mu m}^{2}\right)\left(1\;\text{pS}/\text{ch}\right)}\approx 0.0035\;\sec.=3.5\;\text{ms}$$

Hence, if this 30% smaller membrane is released from *V*_0_ = −20 mV then the time required to reach E is now decreased to 5τ_new_ = 5 (3.5 ms) = 17.5 ms. As a result, in order to conserve the pre‐reduction average discharging rate (i.e., ∆*V*_m_/∆*t* = ∆*V*_m_/[5τ] = −40 mV/25 ms = −1.6 mV/ms), the number of leak channels has to drop by 30% as in doing so the discharging time ∆*t* is preserved since channels density remains unaltered

$$\begin{array}{cc} & \rho_{\text{new}}=\frac{7,000\;\text{ch}}{3,500\;{\mu m}^{2}}=2\;\text{ch}/{\mu m}^{2}=\rho_{\text{old}}\\ & \to \Delta t=5\tau_{\text{new}}=\frac{5{\hat{C}}_{m}}{\rho_{\text{new}}\cdot \gamma }=\frac{5{\hat{C}}_{m}}{\rho_{\text{old}}\cdot \gamma }=5\tau_{\text{old}}=25\;\text{ms} \end{array}$$

Furthermore, the preservation of channels density not only conserves τ and the average discharging rate but also the instantaneous rate d*V*_m_/d*t*. To understand this fact, observe that the above current‐clamp experiment obeys the following initial value problem:

$${\hat{C}}_{m}\cdot \text{Sa}\frac{dV_{m}}{dt}+N\gamma \left(V_{m}‐E\right)=0,\;V_{m}\left(0\right)=V_{0}$$

Thus, the derivative of its solution is

(1) $$\frac{dV_{m}}{dt}=‐\frac{1}{{\hat{C}}_{m}}\left[\gamma \left(V_{0}‐E\right)\right]\left(\frac{N}{\text{Sa}}\right)\exp\left[‐\frac{t}{{\hat{C}}_{m}/\left(\left[N/\text{Sa}\right]\gamma \right)}\right]\;=‐\frac{1}{{\hat{C}}_{m}}u_{\max}\rho \exp\left[‐\frac{t}{{\hat{C}}_{m}/\left(\rho \gamma \right)}\right]$$

where $u_{\max}=\gamma \left(V_{0}‐E\right)$ is the maximal unitary current at the start of the membrane discharge and so the product *u*_max_ *ρ* is the maximal current density. Equation (1) shows that the instantaneous rate at which *V*_m_ decays from *V*_0_ is independent of the size of the cell membrane and is only determined by ρ as long as γ is unaltered. In other words, two passive cells of different sizes but with identical channels types and densities will express the same instantaneous discharge rate when releasing from *V*_0_. Therefore, as a claim in the third sentence of this paragraph, the rate at which *V*_m_ evolves over time is independent of cell size and is ultimately determined by ion channels density.

The above argument can be extended to any membrane with active properties as follows: by analogy to Equation (1) any spontaneously active membrane with *n* different conductance types can be described by the following initial value problem

(2) $$\frac{dV_{m}}{dt}=‐\frac{1}{{\hat{C}}_{m}}\sum_{j=1}^{n}\rho_{j}\cdot u_{j}\left(t,V_{m}\right),\;V_{m}\left(0\right)=V_{0}$$

where *ρ*_j_ is channel density for the j ion channel, while *u*_j_(*t*,*V*_m_) is its unitary current which is some empirically defined the differentiable function of *t* and *V*_m_. The solution to the problem (2) defines the relationship between the rate of spontaneous activity and channel densities. To see why to assume that from *t* = 0 to some other time *t* the membrane potential was continuously displaced from the initial condition *V*_m_(0) = *V*_0_ to *V*_m_(*t*), that is,

$$\int_{V_{0}}^{V_{m}\left(t\right)}\frac{dV_{m}}{dt}dt=‐\frac{1}{{\hat{C}}_{m}}\int_{0}^{t}\sum_{j=1}^{n}\rho_{j}\cdot u_{j}\left(t,V_{m}\left(t\right)\right)dt$$

Hence, the membrane potential at an arbitrary time *t* is given by

(3) $$V_{m}\left(t\right)=‐\frac{1}{{\hat{C}}_{m}}\sum_{j=1}^{n}\rho_{j}\int_{0}^{t}u_{j}\left(t,V_{m}\left(t\right)\right)dt+V_{0}$$

Equation (3) states that in order to reproduce a particular firing behavior that starts from *V*_0_ we are only required to know the set of channels densities $\left\{\rho_{j}|j:=1,2,\ldots ,n\right\}$ and the set of unitary current functions $\left\{u_{j}\left(t,V_{m}\left(t\right)\right)|j:=1,2,\ldots ,n\right\}$; the size of the membrane is unnecessary information. Consequently, two intrinsically active membranes of distinct sizes, but with the same types of ion channels, will show identical autonomous firing patterns if and only if both membranes express the same set of channel densities. Further validation of this conclusion is given by a popular theorem in the field of differential equations: Theorem of the existence of a unique solution (Zill et al., 2013). This theorem postulates that any initial value problem of the form

$$\frac{dV_{m}}{dt}=f\left(t,V_{m}\right),\;V_{m}\left(t_{0}\right)=V_{0}$$

manifests a unique solution if *f*(*t*,*V*_m_) and ∂*f*/∂*V*_m_ are continuous functions in some region of the *t*‐*V_m_* plane that contains the initial condition (*t*_0_, *V*_0_). In problem (2) $f\left(t,V_{m}\right)=‐\frac{1}{{\hat{C}}_{m}}\sum_{j=1}^{N}\rho_{j}\cdot u_{j}\left(t,V_{m}\right)$ and $\frac{\partial f}{\partial V_{m}}=‐\frac{1}{{\hat{C}}_{m}}\sum_{j=1}^{N}\rho_{j}\cdot \frac{\partial u_{j}}{\partial V_{m}}$ are both continuous functions since each *u*_j_(*t*, *V*_m_) is by definition a differentiable function. Hence, Equation (3) is unique and so for a fixed set of unitary current functions it can only be modified whenever the set of channel densities is altered. Therefore, since the answer to the problem (2) is the membrane's pattern of intrinsic activity the statement of unique solution implies that, under cell size reduction, the pretreatment spontaneous spiking behavior is only conserved when the channel density of each conductance is preserved as seen in our simulations.
